# Elective arthroplasty on “high-risk” calendar dates: no evidence for worse outcomes in 43,000 procedures

**DOI:** 10.1007/s00402-026-06405-1

**Published:** 2026-08-22

**Authors:** Nike Walter, David W. Lowenberg, Christian Heiss, Edmund C. Lau, Markus Rupp

**Affiliations:** 1 Department of Trauma, Hand and Reconstructive Surgery, University Hospital Giessen and Marburg GmbH Campus Giessen, Giessen, Germany; 2 Institute of Virology, Helmholtz Center Munich - German Research Centre for Environmental Health, Ingolstädter Landstraße 1, Bavaria 85764 Neuherberg, Germany; 3 Present Address: Department of Orthopedic Surgery, Stanford University, Stanford, USA; 4 Exponent (United States), Menlo Park, USA

**Keywords:** Total hip arthroplasty, Total knee arthroplasty, Revision risk, Mortality, Elective surgical scheduling

## Abstract

**Background:**

Concerns regarding the timing of elective surgery remain common among patients and may influence surgical scheduling. Superstition-associated calendar dates such as Friday the 13th are frequently perceived as “high-risk” time points, yet evidence regarding their impact on arthroplasty outcomes is lacking. This study investigated whether primary total hip arthroplasty (THA) and total knee arthroplasty (TKA) performed on these dates are associated with differences in revision risk, mortality, or procedure frequency.

**Methods:**

A nationwide retrospective cohort study was conducted using Medicare physician service records (2009–2019). Primary THA and TKA procedures were identified in a 5% sample of Medicare beneficiaries aged ≥ 65 years. Outcomes included aseptic revision, septic revision, and mortality. Competing-risk survival analyses (Fine–Gray) and multivariable Cox regression models adjusted for demographic, clinical, and socioeconomic covariates were applied. Procedure volumes were compared between superstition-associated and control dates.

**Results:**

A total of 945 arthroplasties were performed on Fridays falling on the 13th. No significant differences were observed in aseptic revision, septic revision, or mortality compared with control dates. For Friday the 13th, hazard ratios were 0.80 (95% CI 0.44–1.47) for aseptic revision, 0.83 (95% CI 0.31–2.25) for septic revision, and 1.00 (95% CI 0.83–1.19) for mortality. Procedure volumes did not differ significantly between superstition-associated and control dates.

**Conclusion:**

In this large registry-based study, the timing of elective total joint arthroplasty on superstition-associated calendar dates was not associated with increased complication risk, mortality, or changes in procedure volume. These findings may support evidence-based patient counselling and surgical scheduling in elective arthroplasty

## Background

Superstition is a multifaceted concept that involves an erroneous establishment of cause and effect [1]. It has been suggested that individuals turn to superstitious beliefs as coping mechanisms during challenging times, attempting to make sense of and control uncertainty [2, 3]. While superstition may seem at odds with evidence-based healthcare in today’s scientifically dominated world, there is a consensus that surgeons should be mindful of their patients’ spirituality and belief systems [4]. For instance, despite originating more than 2400 years ago, horoscopes and zodiac signs continue to be a constant feature in our society [5, 6]. Forms of superstition, such as considering Friday the 13th as a day of misfortune, are deeply ingrained in the general population [7, 8].

Conducting well-founded investigations into such phenomena could enhance clinician-patient communication and shared decision-making. Therefore, this study aims to address the following research question: Do surgical outcomes, in terms of complication rates and mortality after total knee arthroplasty (TKA) and total hip arthroplasty (THA), get influenced by Friday the 13th? Additionally, as superstitions might affect surgical planning, we have also examined whether there are differences in the number of surgeries performed on these days.

## Methods

In this nationwide cross-sectional study primary hip and knee arthroplasty performed between January 1, 2009 and December 31, 2019 were identified from the Medicare physician service records. These records were compiled by the Centers for Medicare and Medicaid Services (CMS), and after deidentification, were made available for research, known as the Limited Data Set (LDS). Specifically, physician records associated with a 5% sample of Medicare beneficiaries, equivalent to the records from approximately 2.5 million enrolees, formed the basis of this study. The population of interest included elderly Medicare patients (ages 65 and above) who received a primary hip or primary knee arthroplasty during the study period. Since the CMS data is deidentified, it was exempt from review by the Institutional Review Board.

Procedures were identified using Current Procedural Terminology (CPT) codes: 27,125, 27,130, and 27,132 for hip arthroplasty, and 27,446 and 27,447 for knee arthroplasty. Diagnoses were coded using ICD-9-CM (before October 1, 2015) and ICD-10-CM (thereafter). Steps were taken to retain only one record per procedure and to derive laterality from ICD-10 codes, CPT modifiers, or related procedures. Laterality was identified in approximately 95% of arthroplasties.

Three measures were used for evaluation: (a) the frequency of joint arthroplasty performed Friday, the 13th compared to other Fridays, (b) septic and aseptic revision risk for arthroplasty performed on Friday, the 13th and other Fridays (c) survival and mortality of patients with arthroplasty performed on Friday the 13th and other Fridays. The CPT codes used to identify hip revisions were: 27,134, 27,137, 27,138, as well as 27,090 and 27,091. For knee revisions, the CPT codes used were: 27,486 and 27,487, as well as 27,488. Septic revision is defined by having a diagnosis of 996.66 (ICD-9) or T84.5x (ICD-10) indicating infection and inflammation reaction of internal joint prosthesis in the 30 days on or before the date of the revision arthroplasty. The 30-day window was chosen to capture infection diagnoses that were temporally linked to the revision procedure, consistent with established perioperative infection definitions in claims-based research. Revisions without finding any diagnosis indicating infection and inflammation are classified as aseptic. Date of death was identified from the Medicare enrolment data, also provided by the CMS. Individuals were tracked from the arthroplasty date until occurrence of the outcome (revision), until death, until end of enrolment, or until end of study on December 31, 2019, which ever came first.

Survival analysis techniques were used to analyse these outcomes. The Kaplan-Meier (KM) method with the Fine and Gray sub-distribution adaptation was used to calculate the cumulative incidence rate of the septic and aseptic revisions. Septic and aseptic revisions were considered mutually exclusive, and therefore “competing risk” events. For patients undergoing bilateral or staged arthroplasty, each procedure was treated as an independent event, with laterality-specific follow-up for revision outcomes. Multiple procedures per patient were permitted in the analysis. Cases with missing covariate information were excluded from multivariable models. The Fine and Gray technique was used to account for death as a competing event [9]. For mortality or survivorship, the conventional KM method was appropriate. The semiparametric Cox regression was used, also accounting for competing risk, to investigate these outcomes and to compare the risk among patients receiving their arthroplasty on Friday 13th or control dates, after adjusting for a number of potential confounding factors. The demographic factors included: age, sex, race, resident region, and Medicare buy-in (as a surrogate for patient’s economic status). Clinical factors included were osteoporosis, obesity, diabetes mellitus, rheumatoid disease, chronic kidney disease, tobacco dependence, regular use of anti-coagulant, regular NSAID use, prior osteoporotic fracture, hypertensive disease, ischemic heart disease, cerebrovascular disease, COPD, and congestive heart failure. These conditions could appear as either primary or secondary diagnosis, but at least 2 mentions of such condition in the prior year was required to reduce false or “suspect-only” diagnosis. Socioeconomic measures for the patients’ county of residence (median household income, educational attainment, poverty rate, unemployment, urban-rural classification) were obtained from publicly available U.S. Census and federal data sources. All data processing and statistical analyses were performed using the SAS statistical software (Version 9.4, Cary, NC) and significance was determined at α = 0.05.

## Results

### Outcomes after surgeries on Friday, 13th in comparison to other Fridays

**Fig. 1:**
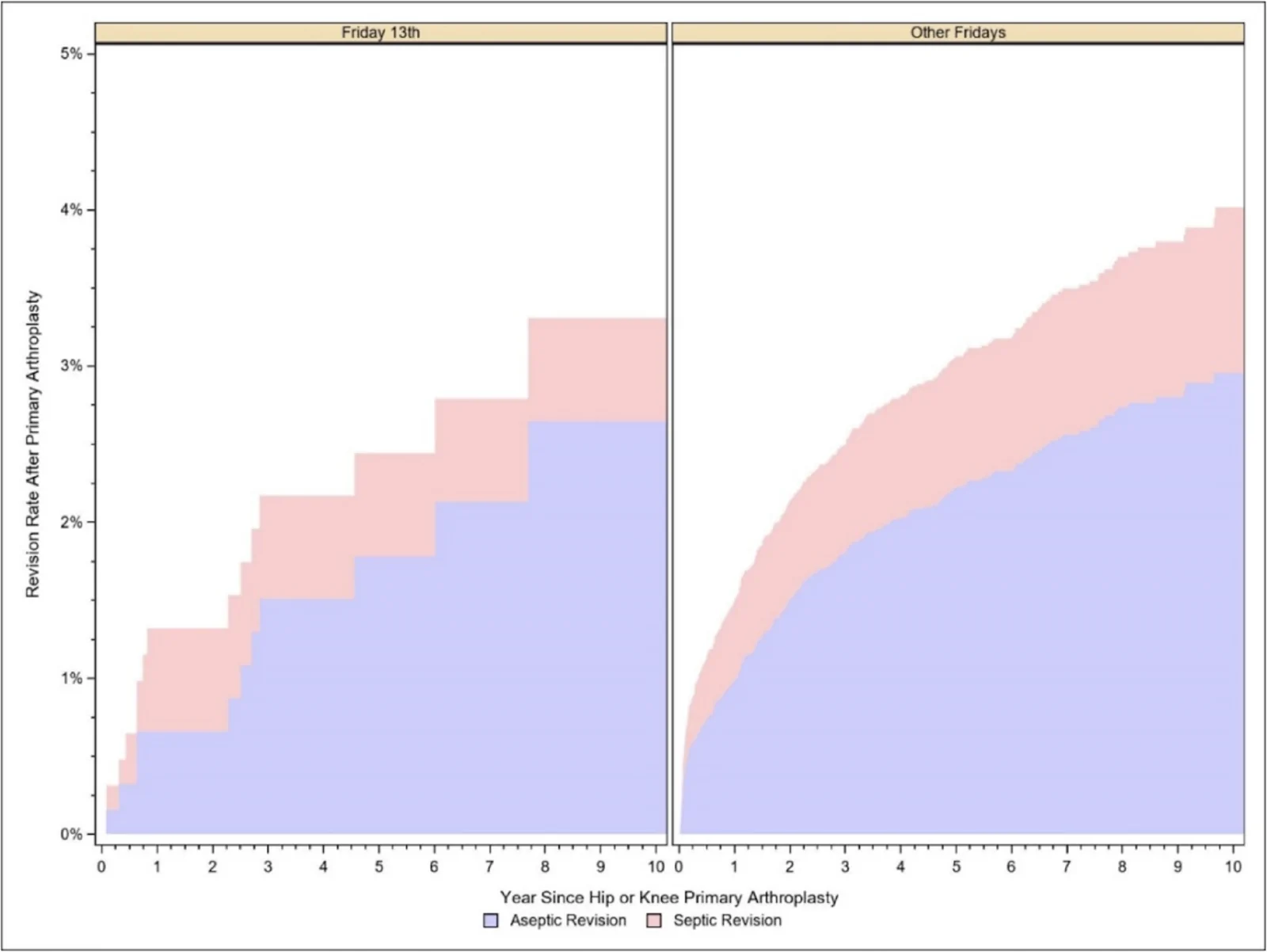
Revision rates after primary hip or knee arthroplasty performed on Fridays, the 13th (left panel) and revision rates after primary THA or TKA performed on other Fridays (right panel). Aseptic revisions are shown in lilac and septic revisions in light pink

**Fig. 2:**
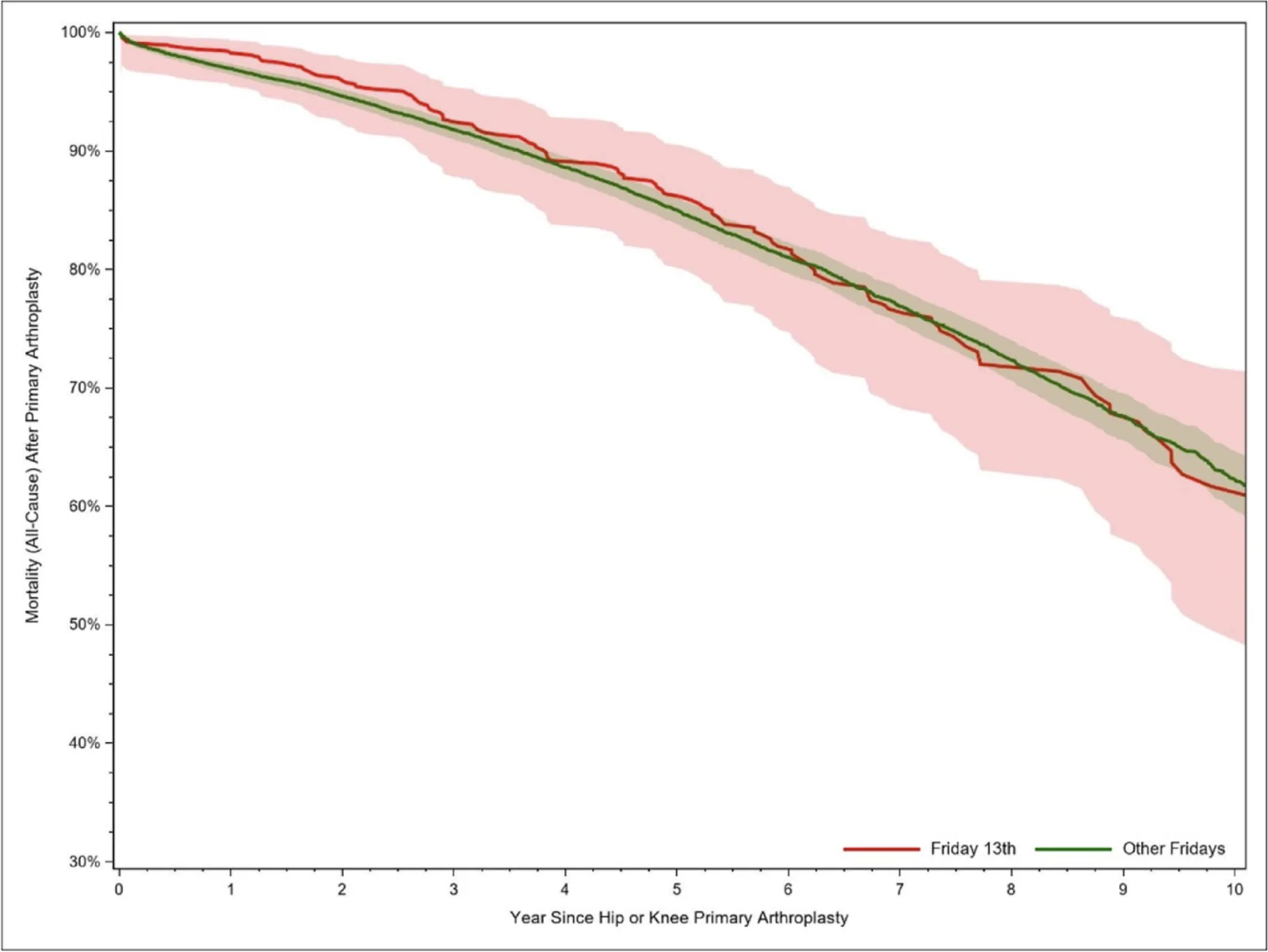
Survival of patients after primary hip or knee arthroplasty performed on Fridays, the 13th and other Fridays

**Fig. 3:**
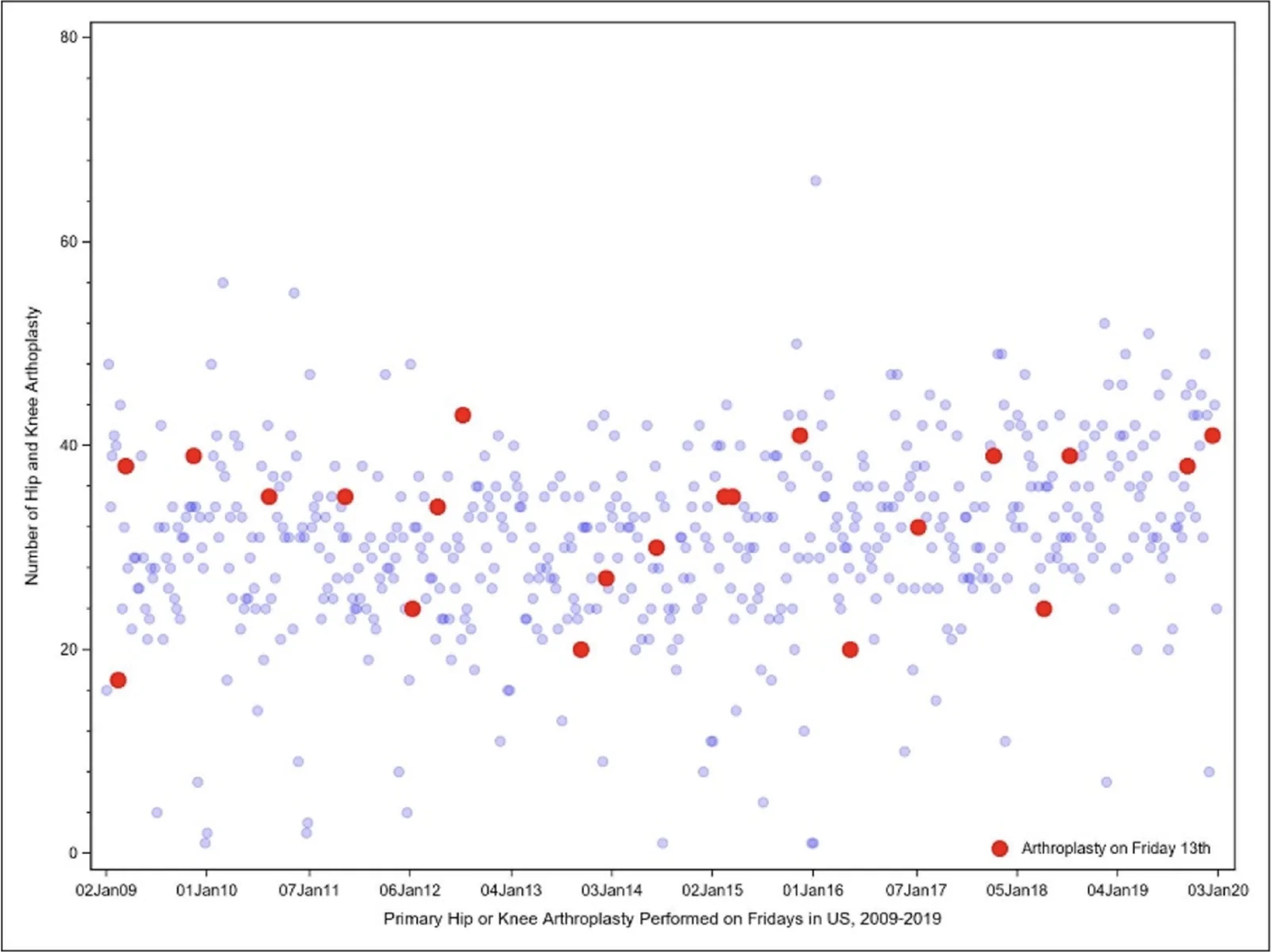
Distribution of primary hip or knee arthroplasty procedures performed on Fridays between 2009 through 2019. Red dots indicate Fridays falling on the 13th

Between 2009 and 2019, 945 arthroplasties (259 THA, 686 TKA) were performed on 21 Fridays falling on the 13th. Five-year cumulative incidence rates for aseptic and septic revisions did not differ significantly between Friday the 13th and other Fridays (Fig. 1). Adjusted hazard ratios confirmed no significant differences: aseptic revision HR 0.80 (95% CI 0.44–1.47, *p* = 0.488), septic revision HR 0.83 (95% CI 0.31–2.25, *p* = 0.726), and mortality HR 1.00 (95% CI 0.83–1.19, *p* = 0.965) (Table 1). The mortality rates did not differ significantly between surgeries performed on Friday the 13th and those on other days (Fig. 2). Additionally, the distribution of procedures performed on Friday the 13th (with a mean of 12.3 ± 4.6 THA and 20.3 ± 4.8 TKA procedures) did not significantly differ from procedures performed on other Fridays (with a mean of 11.5 ± 4.2 THA and 19.4 ± 6.1 TKA surgeries) (*p* = 0.386) (Fig. 3). Moreover, the patient cohorts operated on Friday the 13th and other Fridays did not show statistically significant differences in terms of age and comorbidities, except for a higher proportion of patients using NSAIDs in the other Friday cohort.

**Table 1:** Comparison of risk of aseptic revision, septic revision and mortality after receiving total joint arthroplasty on Friday, the 13th vs. other Fridays

Outcome	HR	95% CI	*p*-value
Aseptic revision	0.80	0.44–1.47	0.488
Septic revision	0.83	0.31–2.25	0.726
Mortality	1.00	0.83–1.19	0.965

*HR* hazard ratio

## Discussion

In this large, nationwide study of Medicare beneficiaries, we found no evidence that elective total hip or knee arthroplasty performed on superstition-associated calendar dates — Friday the 13th or during Mercury retrograde — was associated with increased revision risk, mortality, or changes in procedure volume. Hazard ratios for all outcomes were close to unity, and the results were consistent across joint type, revision type, and analytical approach.

While one might assume that superstition commonly takes a back seat in academic affairs, multiple studies have been conducted on the topic. For example, the “black cloud” phenomenon, referring to surgeons who work unusually harder or handle more complex cases, can still be found in the medical community [10]. Additionally, there exists a belief that uttering the word “quiet” increases the workload in a clinical setting, but no evidence was found to support this in three randomized controlled trials [11–13].

The majority of previously conducted studies have affirmed the findings presented here, indicating no association between perceiving Friday the 13th as a day of misfortune and clinical outcomes. In the field of orthopedics, only one study has thus far addressed this topic. Nardelli and colleagues investigated the pre- and postoperative Western Ontario and MacMaster Universities Osteoarthritis Index (WOMAC) score, and they reported no significant difference in patients undergoing total knee arthroplasty (TKA) on Friday the 13th compared to those operated on other days [14].

Also in other fields, research has shown that Friday the 13th has no influence on intraoperative blood loss and emergency frequency in a study involving 27,914 patients undergoing general, visceral, or vascular surgery [8]. Similarly, in the context of cataract surgeries, Faschinger et al. analyzed outcomes for 16,965 cases and found no statistically significant differences in the number of complications between surgeries performed on Fridays that fell on the 13th and those on other dates [15]. Therefore, the results presented here, based on a large registry database, can provide a sense of certainty and reduce stress for patients who may not feel comfortable or even experience anxiety about undergoing surgery on dates associated with superstitions. This, in turn, could positively influence the healing process [16].

### Limitations

The Medicare data used in this study primarily consists of administrative claims records, which, like any such data, comes with inherent limitations when utilized for orthopedic outcomes research [17, 18]. Notably, we lack access to the detailed clinical records of these patients, and essential clinical measures (such as radiologic imaging findings) and other indicators (like blood chemistry and organ function tests) are not encompassed by the diagnosis or procedure codes in this dataset. Furthermore, important clinical factors such as frailty indices, precise body mass index (BMI) values (beyond the binary obesity diagnosis code), and measures of surgical complexity (e.g., operative time, surgical approach, implant type) were not available in the claims data. While these factors may influence outcomes, there is no reason to expect systematic differences between superstition-associated and control dates, and thus residual confounding is unlikely to bias the comparative findings. While individual records may have occasional errors of omission or commission, it is improbable that systematic errors are present across the millions of records within the entire Medicare system. One significant strength of this data lies in the wide range of facilities submitting these claims and the vast number of records processed by Medicare, providing a unique advantage. Moreover, the extensive information on patient characteristics and complications is of high quality due to its relevance for billing costs and its input by specialized professionals. Additionally, the use of Medicare claims data introduces structural considerations inherent to an insurance-based healthcare system. Healthcare access, utilization patterns, and diagnostic coding are influenced by reimbursement structures, which may introduce selection and information bias. Although such biases are unlikely to differ systematically across superstition-associated and control calendar dates, they may affect absolute risk estimates and limit the generalizability of our findings to universal or non-insurance-based healthcare systems.

## Conclusion

The analysis presented here strongly indicates that the superstitious beliefs of Friday the 13th as a day of misfortune does not have any impact on the outcomes following total hip or knee arthroplasty. These findings can be valuable in enhancing clinician-patient communication, as they address and alleviate potential patient concerns when making decisions about the timing of elective surgeries.

## Data Availability

The data that support the findings of this study are available on request from the corresponding author.
